# Artificial Intelligent Agent Architecture and Clinical Decision-Making in the Healthcare Sector

**DOI:** 10.7759/cureus.64115

**Published:** 2024-07-08

**Authors:** Kian A Huang, Haris K Choudhary, Paul C Kuo

**Affiliations:** 1 Surgery, University of South Florida Health Morsani College of Medicine, Tampa, USA

**Keywords:** cognition, ai, physician decision-making, physician metacognition, intelligent agents

## Abstract

This paper examines the decision-making processes of physicians and intelligent agents within the healthcare sector, particularly focusing on their characteristics, architectures, and approaches. We provide a theoretical insight into the evolving role of artificial intelligence (AI) in healthcare, emphasizing its potential to address various healthcare challenges. Defining features of intelligent agents are explored, including their perceptual abilities and behavioral properties, alongside their architectural frameworks, ranging from reflex-based to general learning agents, and contrasted with the rational decision-making structure employed by physicians. Through data collection, hypothesis generation, testing, and reflection, physicians exhibit a nuanced approach informed by adaptability and contextual understanding. A comparative analysis between intelligent agents and physicians reveals both similarities and disparities, particularly in adaptability and contextual comprehension. While intelligent agents offer promise in enhancing clinical decisions, challenges with types of dataset biases pose significant hurdles. Informing and educating physicians about AI concepts can build trust and transparency in intelligent programs. Such efforts aim to leverage the strengths of both human and AI toward improving healthcare delivery and outcomes.

## Introduction

With recent developments in artificial intelligence (AI), such as ChatGPT, computers, programs, and robots are evolving to think more like humans than ever before. Academic papers studying AI grew by more than 400% from 1996 to 2017, illustrating a collective interest in this trending field [1]. Regarding the private sector, from 2000 to 2017, AI start-ups have increased 14-fold, venture capital investments in AI industries have increased six-fold, and robot imports into North America have increased 2.5-fold [1]. Everyday people likely interact with AI daily, as large companies such as Amazon, Google, and Netflix have fine-tuned AI algorithms to support consumer purchase and consumption [2]. As AI grows in capability and demand, it is crucial to understand how it will impact the healthcare field. 

With the rise of total medical costs and the increasing scarcity of physicians, healthcare is a promising field for AI support to improve efficiency and throughput. Drug discovery, clinical trial research, and medical imaging recognition are just a few of the many large-scale opportunities in healthcare that have already been implemented in AI [2]. Recent research has also proposed roles AI could fulfill that work more closely with physicians and at the forefront of healthcare, specifically in clinical decision support. For example, when prescribing antibiotics to patients, doctors need to be vigilant about preventing multidrug-resistant diseases; an AI system that considers a range of variables from patient and hospital data could help reduce the overuse of antibiotic treatment [3]. More concretely, AI programs have been shown to have safer triage and diagnosis decision-making skills comparable to physicians in controlled patient simulations and offer valuable information to improve clinical decision support in hypothetical medical scenarios [4,5]. As AI continues to grow and infiltrate healthcare, it becomes crucial that physicians have a general idea of how an AI program, or “intelligent agent,” makes decisions.

## Technical report

Intelligent agents

What Are Intelligent Agents

**Figure 1:**
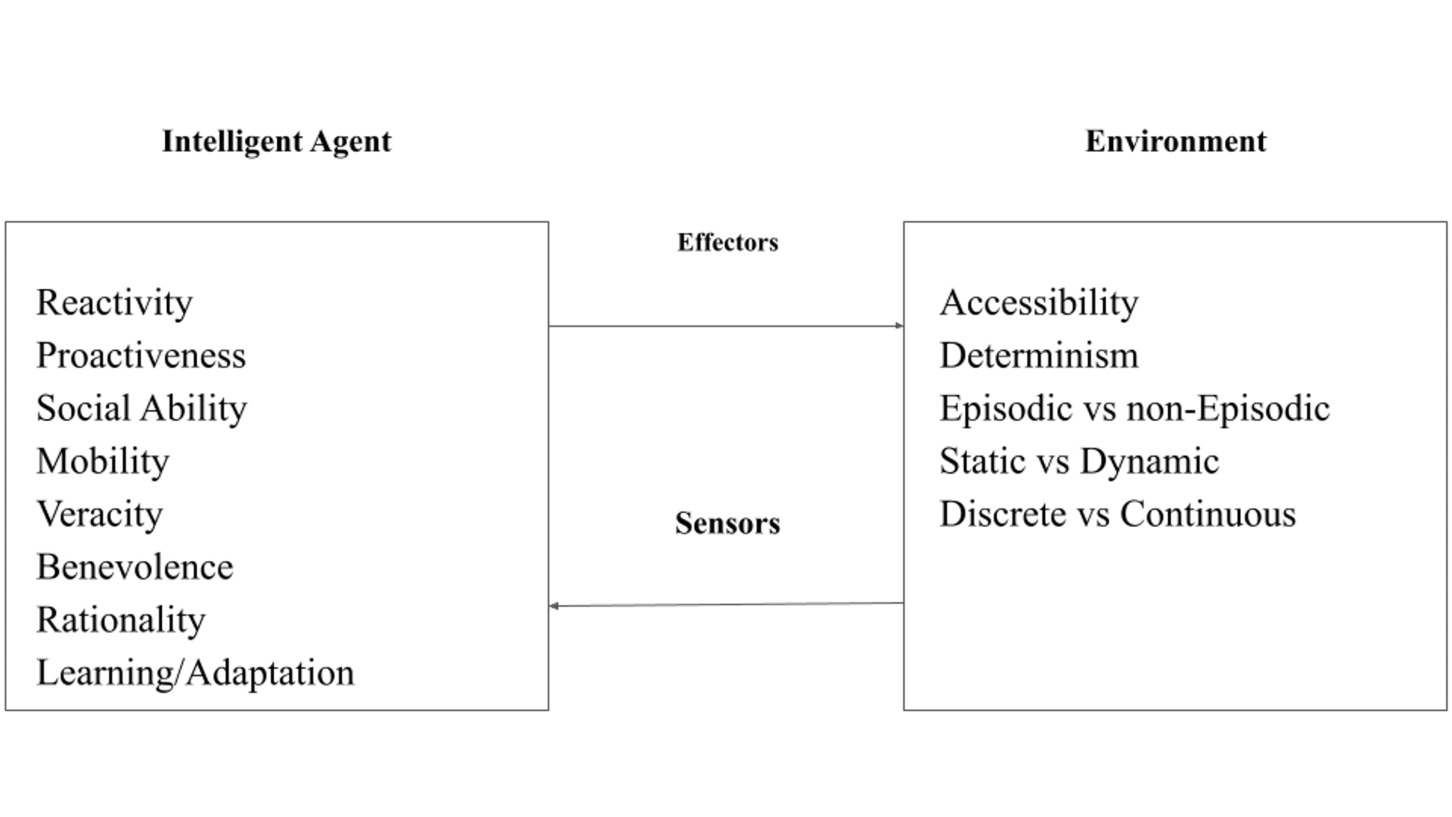
Properties between an intelligent agent and its environment Visual representation of the dynamic relationship between an Intelligent agent and its environment and their respective traits that may modify such relationship. Image credit: [7]

Intelligent agents can be defined as independent software or hardware entities that can perceive their environment through sensors, collect information, process it, and choose to react to it through effectors or actuators to achieve a desired goal or outcome [6,7]. This process is illustrated in Figure 1. In the simplest example, an agent could be compared to a thermostat, constantly perceiving temperature throughout its environment (via sensors), comparing this value to a predetermined range of reference values, and conditionally using its inherent function to enact a change in temperature upon its environment (via effectors). 

Agent-Based Properties 

Agent-based properties can modulate an agent’s disposition to observe and behave toward its environment including the following properties [6,7].

Reactivity: An agent can perceive and respond to changes or stimuli in its environment in real-time. It involves the agent's ability to react promptly to incoming data or changes, without necessarily relying on prior planning or goal-setting.

Proactivity: An agent's capacity to take initiative and exhibit goal-directed behavior without external stimulation or requests, involves the agent's capability to anticipate future events or changes, plan actions, and act in pursuit of its goals without waiting for explicit commands.

Social ability: An agent's skill in interacting, communicating, and collaborating with other agents or entities in its environment involves the agent's capability to engage in cooperative behavior and effectively communicate or negotiate with other agents. An example would be a fleet of drones in a drone light show that confer aerial positioning to one another to synchronize their movements without collision. 

Mobility: An agent's ability to move or operate in different physical or digital environments involves the agent's capability to transition between different locations or platforms to perform tasks, gather information, or achieve its objectives.

Veracity: Trustworthiness, accuracy, and reliability of the information an intelligent agent receives, processes, or provides. It involves ensuring that the data used by the agent is reliable, free from errors or biases, and accurately reflects the real-world scenario.

Benevolence: The ethical aspect of an intelligent agent's behavior. It denotes the agent's intention to act in a morally good or beneficial manner, considering the well-being of others, and adhering to ethical principles or guidelines in its actions and decisions.

Rationality: An agent’s ability to make logically sound decisions and choices based on available information and their objectives or goals involves assessing alternatives, weighing consequences, and selecting the most optimal or rational course of action given the circumstances.

These properties collectively define various aspects of an intelligent agent's capabilities, ranging from its responsiveness to the environment, ethical considerations, social interactions, decision-making, and adaptability in different situations.

Agent Environment-Based Properties

Environment-based properties discern the complexity of the physical or digital habitat the agent must map, interact with, and process while pursuing its goal [6,7].

Accessibility: An agent's ability to obtain information about its environment. An environment is considered accessible if the agent can obtain complete and accurate information about the environment's state at any given time.

Determinism: The predictability of an environment's behavior is based on the agent's actions. A deterministic environment means that, given a particular state and action, the next state of the environment is always precisely determined and predictable.

Episodic: The agent's experience is divided into discrete episodes or episodes that do not affect each other. Each episode is independent, and the agent's actions in one episode do not influence subsequent episodes.

Non-episodic: The agent's actions or experiences in one episode can affect or influence future episodes. The current state and actions can impact future states and actions of the agent.

Static: The environment does not change while the agent deliberates its actions. The environment remains constant throughout the agent's decision-making process.

Dynamic: The environment can change even when the agent is not acting. Changes occur independent of the agent's actions and affect the environment's state.

Discrete: An environment with a finite and well-defined set of distinct states. Transitions between states are well-defined and do not involve intermediate or continuous values.

Continuous: An environment with an infinite number of possible states, or the state space, is continuous. Transitions between states involve continuous values or have a vast number of possible states without clear boundaries.

These properties characterize the nature of environments in which intelligent agents reside, with the real physical world being the most complex environment in which agents can independently operate.

Intelligent Agent Architectures

Intelligent agent architectures are frameworks that define the structure and behavior of agents in AI systems. They can be divided into four main types [8,9]: 

Reflex-based agents (RAs): As the name delineates, RAs reflexively select and execute actions based on the current "percept" (information received from the environment), highlighting the aforementioned agent-based concept of reactivity. RAs typically follow "if-then" rules (predefined rules or conditions), mapping specific percepts to actions. A consequence of this architecture is that these agents do not consider the consequences of their actions beyond the immediate perception; hence, they might not be suitable for complex environments. There are two types of RAs: simple RAs and model RAs. A simple RA is characterized by making decisions based solely on its current percept and lacks the ability to learn from its previous experiences and interactions - often used due to its immediate processing speed, providing fast feedback [8,9]. A real-world representation of a simple RA could be a stereotypical consumer-grade vacuum cleaning robot [8,9]. A model RA is a slightly more refined and complex version of a simple RA, with the ability to keep track of the state of its environment as a model based on historical percepts. It understands that the environment around them may change independently of the effects of their actions, often having an additional (but limited) set of decision-making conditions and rules to augment or enhance delusional outcomes [8,9]. 

Goal-based agents (GAs): GAs have an internal goal representation that guides their behavior and considers the future consequences of their actions toward their goals [8,9]. This necessitates the calculation of a long sequence of actions required to reach their goal, instead of the immediate anticipation of a single action [8,9]. GAs are flexible agents, as the reasoning for their decisions can be represented in a deliberate and meaningful way that can be modified by changing their goal rather than a reflexive "if-then" instinct. Unlike an RA, GAs favor the agent-based property of proactivity over reactivity, typically conferring a greater degree of autonomy through the ability to self-initiate a precalculated string of actions to achieve a desired goal without a preceding stimulus. 

Utility-based agents (UAs): UAs make decisions beyond the binary measure of whether or not a goal was achieved. Instead, UAs evaluate how the goal can be achieved, as not all paths to the same goal are equally reliable, efficient, economical, quick, or safe. Rather than aiming to achieve specific goals, they consider trade-offs between competing goals or outcomes, calculate the expected usefulness or 'utility' of various actions, and choose the one that maximizes the overall utility [8,9]. Distinct from a GA, UAs rely more heavily on the property of rationality, weighing the marginal gains and losses of different paths of actions to take, which is critical when improving an intelligent model through optimization. 

**Figure 2:**
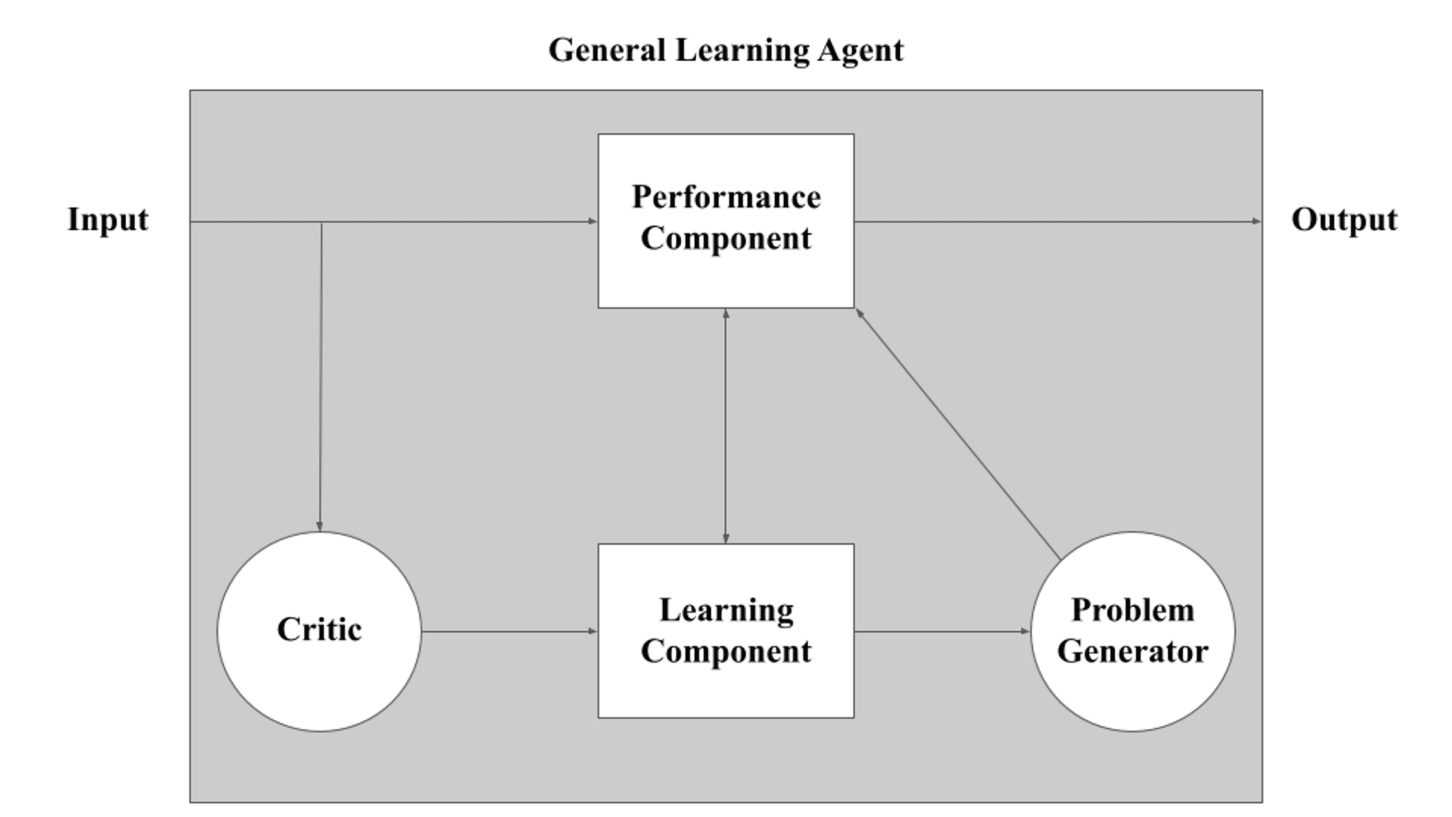
General learning agent model Visualization of an architectural schematic for a general learning agent. Image credit: [8]

General learning agents (GLAs): As shown in Figure 2, GLAs are derived from four main components: the performance element, the learning element, the critic, and the problem generator [8,9]. The performance element decides which actions to execute in the environment based on its percepts - what was previously described as the entirety of an intelligent agent (RAs, GAs, and UAs). The learning element measures the execution and completion of the performance element and makes changes that aim to improve performance in the future. The learning element does this by collecting feedback from the critic, which is a component that compares the performance element to a preprogrammed performance standard, allowing the agent to understand how successful it was in completing its task. Lastly, the problem generator challenges the performance element in undertaking and exploring new tasks, potentially discovering new strategies for new problems to adapt to novel situations, acquire knowledge, and refine their behavior. It should be noted that GLAs are not a discrete type of agent, and RAs, GAs, and UAs can be designed as GLAs.

Each architecture has its strengths and weaknesses, making them suitable for different environments and tasks. For instance, simple reflex agents are straightforward but limited in complex environments. GAs are more adaptive and goal-oriented, UAs consider trade-offs, and GLAs continually improve based on experience. The choice of architecture depends on the specific requirements and characteristics of the problem. For example, algorithms measuring the EEGs and EMGs in subjects need to be quick and straightforward - a task that lends itself to a simple RA [9]. However, an agent that can learn, improve, and adapt to novel and complex clinical presentations will be the most suitable option to make a more nuanced and multifaceted decision or to support physicians making those decisions. 

Physician clinical decision-making

Clinical Decision-Making Structure of Physicians

Physicians can be considered to use one of two general groups of cognitive strategies when making a clinical decision: intuitively automatic experiential-driven reasoning (experiential) and a more rationally conscious and logic-based type of reasoning (rational) [10-12]. Experiential decision-making is rooted in experience, intuition, and heuristics that enable a physician to create a rapid clinical decision with acceptable utility, which can be invaluable in time-sensitive life-saving cases. Contrastingly, rational decision-making typically involves analyzing empirical data, referencing clinical guidelines, and consciously gathering and evaluating all variables to reach a decision. Given that practicing physicians tend to prefer rational decision-making, and that experiential decision-making is a uniquely human process not comparable to intelligent agents, this paper will focus on the rational side of physician decision-making [10,11].

**Figure 3:**
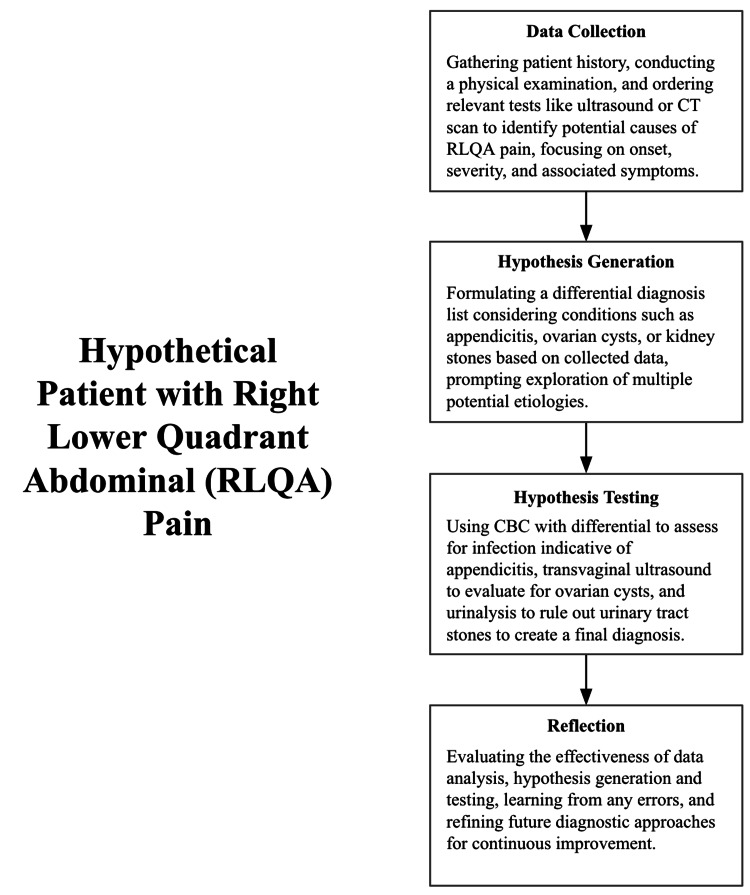
Physician rational decision-making Hypothetical patient scenario showcasing a physician’s rational clinical decision-making process. Image credit: [12]

As seen in Figure 3, rational decision-making preceding diagnosis, as articulated by Trimble and Hamilton, can be modeled in four steps: data collection, hypothesis generation, hypothesis testing, and reflection. Data collection involves gathering medical information on a patient (lab values, medical history, physical examination, etc.) relevant to the support of a diagnosis [12]. This information is then processed, interpreted, and weighed for significance to analyze patterns and inconsistencies in the patient data to prepare a differential diagnosis, which leads to the next step. Hypothesis generation is akin to creating a list of possible diagnoses based on the information collected, ranging from most to least probable. The purpose of creating a differential is twofold. It both coerces the process of considering multiple possible diagnoses and protects against a range of biases that may corrupt the decision-making process, such as anchoring to a particular diagnosis based on initial evidence and keeping it beyond its total merit [13]. Once a differential is synthesized, the next step proceeds. In hypothesis testing, the ideas are scanned for inconsistencies and patterns of relevant discerning characteristics to isolate the initial batch of hypotheses to be tested [12]. Then diagnostic tests are used to confirm or refute possible hypotheses, requiring effortful and meticulous thought, both in interpreting the information to create a likely candidate diagnosis and conscious monitoring of unexpected results with the willingness to reassess the current diagnosis [12]. Cognitive strategies, such as deduction, induction, abduction, rule-based reasoning, probabilistic reasoning, and causal reasoning, can also help physicians refute or affirm potential diagnoses (Table 1). The final step, reflection, helps serve as feedback to evaluate the efficacy and quality of the physician’s final diagnosis. If an error occurs during or after the clinical decision, it gives the physician time to think about what went wrong, re-evaluate their thinking process to look at potential points of failure, and future-proof their clinical reasoning process by learning from their mistakes and adjusting their cognition accordingly. Ultimately, reflection is a form of self-monitoring that allows the physician to catch and understand mistakes in their reasoning and improve over time.

**Table 1:** Modes of clinical decision reasoning Presented are tabulated modes of reasoning commonly used by physicians during the clinical decision-making process. Each type of reasoning is categorized with a description and a hypothetical example. Table credit: [12,13]

Mode of reasoning	Description	Example
Deductive reasoning	Deriving specific conclusions from general principles or premises	If a medical textbook states that fever (A) is always caused by an infection (B), and a patient has a fever (A), then deductive reasoning would conclude that the patient likely has an infection (B).
Inductive reasoning	Inferring general principles or conclusions from specific observations or examples	After observing multiple patients with a specific set of symptoms (A) who all test positive for a certain virus (B), one might inductively generalize that these symptoms indicate the presence of that virus in similar cases.
Abductive reasoning	Forming the most likely explanation or hypothesis based on the available evidence	A patient presents with cough and shortness of breath (A). Abductive reasoning might suggest that the most likely explanation is pneumonia (B) based on common causes of these symptoms.
Rule-based reasoning	Applying predefined rules or principles to make logical decisions or draw conclusions	If a medical guideline prescribes a specific treatment (B) whenever a patient has a certain condition (A), rule-based reasoning would lead to the conclusion that the patient with that condition should receive the prescribed treatment.
Probabilistic reasoning	Assessing the probability of different outcomes based on available information	Assessing the probability that a patient with a history of smoking (A) will develop lung cancer (B) based on statistical data and risk factors associated with smoking.
Causal reasoning	Identifying cause-and-effect relationships between events or variables	Identifying that exposure to a particular toxin (A) is causally linked to the development of a specific disease (B), establishing a cause-and-effect relationship between the two in a medical context.

Of course, a discussion of physician cognition would be incomplete without mention of unrecognized biases that impact their ability to make a decision. Unlike intelligent agents, the human mind is fallible in a way that is hard to monitor, as unconscious processes may plague the decision-making process at any point of our multi-step data collection, hypothesis generation, hypothesis testing, and reflection framework. Cognitive errors are prevalent in clinical practice, with up to 75% of errors in internal medicine being cognitive in origin [14]. A systematic review conducted by Saposnik et al. analyzed 20 publications involving 6,810 physicians to identify some of the most common forms of cognitive biases. The study found overconfidence, anchoring, information, and availability bias to be the most common biases of the 19 studies [15]. Overconfidence bias manifests when physicians exhibit excessive certainty in their diagnoses or treatment plans, leading to diagnostic inaccuracies and inappropriate management decisions. The review found overconfidence to have a prevalence between 50% and 70% of participants [15]. The anchoring effect occurs when physicians fixate on initial information and fail to adjust adequately when new information becomes available. For example, a physician may get anchored to an initial diagnostic impression and disregard contradictory evidence, leading to errors. The prevalence of anchoring bias among physicians ranged from 5.9% to 87.8% [15]. Both information and availability biases can contribute to diagnostic errors by causing physicians to overlook or undervalue relevant information. Information bias refers to the tendency to seek out and assign more weight to information that confirms one's preexisting beliefs or hypotheses while discounting contradictory evidence; whereas, availability bias involves judging the likelihood of an event based on how easily examples come to mind, often influenced by recent or memorable cases rather than empirical data [14]. The prevalence of availability bias among physicians ranged from 7.8% to 75.6% [15].

## Discussion

Comparing intelligent agents and physicians in rational decision-making 

Physician decision-making is algorithmic, involving discrete steps for information gathering, hypothesis generation, testing, and reflection [12]. GLAs also have a similar paradigm: gathering objective data from the outside world using sensors, comparing the collected data to internal precepts and objectives, executing an action using an appropriate effector, and reflecting on its performance using self-created feedback and performance standards. These shared steps in meta-cognition lend GLAs well to assist physicians in decision-making, although there are a few differences to delineate. 

Physicians are highly adaptable, using their judgment to switch between various modes of reasoning (Table 1) to accommodate novel or complex cases and adjust their approach based on the uniqueness of each patient. In contrast, the adaptability of intelligent agents varies based on their architecture, with GA, UA, and GLA being more adaptable than a simple RA. GLAs contain the highest capacity to adapt, having the ability, albeit limited, to self-monitor their performance and challenge themselves with simulated problems and obstacles. This skill is imperative in the medical environment, as patients with similar conditions may present variably and with concomitant pathologies, and multiple or different strategies may be required to encapsulate and interpret patient data and create a diagnosis. Generally, most agents are only able to complete tasks that they were explicitly trained to do with the dataset that they were trained on (e.g., an agent that visualizes chest X-rays may not have the capacity to read MRIs, CTs, or ultrasounds, and an agent used to process organized and structured data may be useless in interpreting unstructured or informal clinical notes written by healthcare practitioners). With regard to the advent of large language models (LLMs) such as ChatGPT, which has demonstrated clinical utility in a wide array of simulated patient encounters, its depth of knowledge, quality, and scope is still confined to the boundaries of the data - human literature in this case - fed into it during its training and maturation [5]. This also leads to the next critique of intelligent agents regarding dataset bias.

Physicians excel at contextual understanding, meticulously considering individual patient histories, previous hospitalizations, concurrent medications, and sociocultural factors to tailor their treatment approach. In contrast, intelligent agents face inherent limitations dictated by the quality and diversity of the datasets used to train them. This phenomenon is known as dataset bias, where AI programs demonstrate poor generalizability beyond the original datasets used during training [16]. Even if an agent were to capture the richness of patient context, it must contend with the assumption that the patient encounter aligns with the distribution of observations within training datasets. Moreover, ensuring that training data accurately represents the diversity of patient populations poses significant challenges, potentially leading to biases and disparities in AI-driven healthcare applications. For example, studies on gender, age, and facial recognition have shown that AI algorithms trained on imbalanced datasets may exhibit racial or gender biases, resulting in inequitable outcomes for certain demographic groups [17,18]. In these cases, there was representation bias - a subtype of dataset bias - where an under-representation of faces from certain genders and ethnicities (East Asian, African American, etc.) and an over-representation of Caucasian data points caused models to perform poorly in recognizing the former and to excel in recognizing the latter. 

Another type of dataset bias is domain shift bias, where there is a gradually growing difference between the data in training and the data in deployment (the domain in which the model will be used). Domain shift bias can arise for various reasons, such as changes in the environment, differences in data collection procedures, or shifts in user behavior over time [17]. Ultimately, this difference in distribution can lead to degraded performance or unexpected behavior of the model when applied in the real world [19]. An example of domain shift bias would be the hypothetical development of a dog breed identification model that uses a dataset of professionally photographed dog breeds. As the model is integrated into a phone application for pet owners, it struggles to accurately classify user-uploaded images due to variances in quality, lighting conditions, backgrounds, and poses compared to the professionally curated dataset used for training. Therefore, unlike physicians, current intelligent agent systems may wrestle with the ability to appropriately generalize to new data outside of their training datasets, highlighting a crucial limitation, given that patient backgrounds may drastically differ day-to-day within clinical encounters.

Future role of intelligent agents in clinical decision-making 

The landscape of healthcare is complex and ever-changing. As large quantities of digitized patient data begin to surge, the training, development, and application of clinical decision-making agents become more viable. As more AI-driven programs in fields such as dermatology, neurology, and pediatrics are experimentally validated and match or even outperform experienced physicians, it becomes imperative that physicians gain insight into how intelligent agents function in general [20]. This is because physicians who grasp the framework of the programs that they will eventually operate in a clinical setting are better equipped to critically evaluate the outputs produced by these systems. They can assess the validity, reliability, and potential biases of AI recommendations, ensuring that patient care decisions are based on sound evidence and reasoning. Furthermore, building a healthy understanding of intelligent agent function allows physicians to build trust and transparency and negate a “black box” effect - where the underlying mechanisms of a system are not transparent or comprehensible to the user - that may grow skepticism against clinical decision support programs, creating a larger barrier against the adoption of such systems. Therefore, future physician education should aim to include foundational concepts of agent structure and basic concepts of AI reasoning.

Despite the comprehensive examination of intelligent agents and their potential role in healthcare, this study has several limitations that should be acknowledged. The study relies on a selected range of academic papers and publications, which may not encompass all relevant research, potentially resulting in an incomplete overview of the latest advancements. The findings, based on existing literature and theoretical frameworks, may not fully capture the variability and complexity of real-world clinical settings, limiting their generalizability to diverse healthcare environments and patient populations. Additionally, while discussing the potential capabilities of intelligent agents, the study lacks empirical validation through practical experiments or clinical trials, making the practical applicability and effectiveness of these agents in actual healthcare settings speculative. Technological limitations, such as computational constraints and algorithmic inefficiencies, are not deeply explored, and ethical considerations are only briefly touched upon. Furthermore, the rapidly evolving nature of AI research necessitates continuous updates to maintain relevance and accuracy. Future research should address these limitations by incorporating a broader range of data sources, conducting empirical validations, and exploring ethical and technological challenges in greater detail.

## Conclusions

As AI becomes increasingly ingrained in healthcare, understanding the roles of intelligent agents and physicians is paramount. While intelligent agents offer the potential for augmenting clinical decisions, they face challenges such as dataset bias, contrasting with the adaptability and contextual understanding of physicians. Moving forward, integrating the strengths of intelligent agents with the expertise of healthcare professionals holds promise for improving diagnostic accuracy and patient outcomes. This integration necessitates embedding AI concepts into medical education and fostering trust between physicians and AI systems. Collaboration across disciplines is crucial for ensuring ethical AI development and deployment in healthcare. By prioritizing equity and patient-centered care, the fusion of human and AI can propel healthcare delivery into a new era of effectiveness and efficiency.
